# Prevalence and influence factors of occupational exposure to blood and body fluids in registered Chinese nurses: a national cross-sectional study

**DOI:** 10.1186/s12912-022-01090-y

**Published:** 2022-11-04

**Authors:** Lihui Zhang, Qi Li, Ling Guan, Lu Fan, Yunxia Li, Zhiyun Zhang, Sue Yuan

**Affiliations:** 1 Teaching and Research Section of Clinical Nursing, Xiangya Hospital of Central South University, Changsha, 410008 China; 2 Xiangya Nursing School, Central South University, Changsha, 410013 China; 3 Nursing Department, Beijing Ditan Hospital of Capital Medical University, Beijing, 100015 China

**Keywords:** Bloodborne pathogens, Cross-sectional study, Needlestick, Nurses, Occupational exposure

## Abstract

**Background:**

Occupational exposure to blood and body fluids poses a threat to medical providers and to nurses especially. This harm is not only physical, but psychology as well and can ultimately impact patient safety. This study aims to understand the prevalence of occupational exposure to blood and body fluids among Chinese registered nurses and explores the factors that influence this exposure.

**Methods:**

A cross-sectional online survey was conducted for 31 province-level divisions in China, using a self-created questionnaire entitled *Status Survey on Occupational Exposure in Nurses*. Descriptive statistics were used to describe both the demographic characteristics of the respondents and the characteristics of occupational exposure. Categorical variables were presented as frequencies and percentage, and the relationship between possible influential factors and the occurrence of occupational exposure was determined using binary logistic regression.

**Results:**

Out of a total of 20,791 nurses analyzed, over half (52.1%) of them had experienced occupational exposure to blood or body fluids, but over 1/3 (34.6%) of them did not ever report their exposures to a supervisor/official. The top three causes of under-reporting were: the source patient failed to test positive for infectious pathogens (43.6%), perception of a burdensome reporting process (24.6%), and indifferent attitude towards being infected (16.9%). Nurses who worked over 8 hours per day had higher risks of exposure (OR 1.199, 95% CI 1.130 to 1.272, *P* < 0.001, respectively). The occupational exposure risk from providing 1–2 types of PPE is 1.947 times that of providing 9–10 types of PPE (OR 1.947, 95% CI 1.740 to 2.178, *P* < 0.001). Likewise, the occupational exposure risk of providing 1–2 types of safety-engineered injection devices is 1.275 times of that of providing 5–6 types (OR 1.275, 95% CI 1.179 to 1.379, *P* < 0.001).

**Conclusions:**

Occupational exposure to blood and body fluids in registered nurses is common, but the rate of under-reporting such exposure is high. Implementing engineered “sharp” injury prevention devices, following exposure prevention procedures, giving sufficient education and training to healthcare personnel on exposure prevention and control, and developing exposure reporting policies are all steps that can both reduce exposure and increase its reporting.

**Supplementary Information:**

The online version contains supplementary material available at 10.1186/s12912-022-01090-y.

## Introduction

Occupational exposure to blood and body fluids is when healthcare providers come into contact with potentially infectious blood or body fluids when performing their job duties. Exposure can occur via broken skin or mucous membranes exposure, or when skin is pierced by a contaminated sharp instrument (a “sharp”) [1, 2]. Nurses in particular are more likely to be exposed to bloodborne pathogens due to their more frequent and closer contact with patients than other types of providers such as physicians.

These pathogens can be relatively harmless, or quite serious, as is the case with Hepatitis B Virus (HBV), Hepatitis C Virus (HCV), and Human Immunodeficiency Virus (HIV) [3–5]. The World Health Organization (WHO) has reported that approximately 3 in 35 (3 million / 35 million) healthcare providers worldwide experience percutaneous exposure to bloodborne pathogens annually, and of these providers, two million healthcare providers suffered occupational exposure to HBV, 900,000 to HCV, and 170,000 to HIV, which had the potential to cause 70,000 HBV, 15,000 HCV, and 500 HIV infections as a rough estimate [6]. Needlestick injuries are the most common type of occupational exposure to blood and body fluids [7]. A cross-sectional survey conducted at 81 hospitals in Shanghai showed that 1.53% of healthcare providers had experienced at least one sharp injury in the past month [8].

Further studies have shown that occupational exposures can bring physical harm to healthcare providers, trigger psychological concerns such as anxiety, fear, stress, and insomnia [9–11], and affect their satisfaction with their jobs [12]. Moreover, all of these consequences for nurses are potentially detrimental to patient safety as well [9, 13]. In addition, occupational exposure increases the costs of providing medical care. Previous studies have shown that the total direct and indirect costs associated with drug toxicity and lost time from work from a single exposure event to HBV, HCV, and HIV are RMB 5936, RMB 5738, and RMB 12,709 respectively [14]. Another study reported that the estimated direct cost of a needlestick injury associated with an insulin injection was between RMB 1884 and 2389 [15].

To strengthen occupational exposure management of healthcare providers, many countries have issued guidelines related to occupational exposure [16–18] that involve pre-exposure prevention, immediate treatment, post-exposure prophylaxis (PEP), and follow-up. To reduce the risk of needle-stick injuries during patient care, the WHO even called for worldwide use of safety-engineered syringes by 2020 [19] and proposed “Health worker safety: a priority for patient safety” as the theme for World Patient Safety Day 2020 [13]. The International Labor Organization (ILO) has also taken aim at lowering occupational exposure for nurses by providing protective equipment, following the operating procedures, using safety instruments and creating a relatively safe environment [20].

However, there are still many healthcare providers who do not follow the guidelines and recommended operating procedures [1, 10, 21]. One survey showed that over 40% of healthcare providers recap of the used needles [1]. Additionally, over 70% of providers reported a shortage of personal protective equipment (PPE), and over 80% stated that they did not always wear eye goggles when there was splash risk of blood and body fluids [1]. Clearly, continuing to explore the prevalence and influencing factors associated with occupational exposure to bloodborne pathogens remains of great importance.

Compared to other countries, the English-language literature from China is still insufficient [22]. Most of the previous studies on the prevalence of occupational exposure have been based on reported data focused on only a certain type of occupational exposures, such as needlestick, and there are few studies on nurses in particular with large samples [23–25]. Thus, this paper fills what is currently an unfortunate gap in the literature. Specifically, this study aims to understand the current situation of occupational exposure to blood and body fluids among Chinese nurses using a cross-sectional analysis, and to analyze the factors that influence this exposure using logistic regression.

## Method

### Study design and participants

The data for this come from a survey that was conducted with nurses in 31 province-level divisions (22 provinces, 4 municipalities, and 5 autonomous regions) in mainland China. We used convenience sampling, to select a sample whose size was calculated according to the formula:

$$\mathrm n\frac{\mathrm u_{\alpha/2}^2\pi\left(1-\pi\right)}{\delta^2}$$

This formula is often used in cross-sectional studies to calculate sample sizes. Here, α was 0.05, u was 1.96, π was 50%, and δ was 0.01 so that n found to be to 9604, but the actual sample size ended up being much larger than this. Participants cover four economic regions of China, eastern, middle, western, and northeastern, and for each region the number of nurses in our study exceeded 3/1000 of the total number of nurses in the region in 2019. Therefore, the sample is likely to be a good representation of the overall situation in mainland China.

We administered our questionnaire through the online survey platform Questionnaire Star. Using information from the Chinese Nursing Association, questionnaire links were sent to the members of the special committee who then arranged for the staffs of medical institutions in the region to complete the questionnaires. Nurses filled out the survey by clicking on the survey link or the Quick Response Code of the questionnaires, which were forwarded by the healthcare provider working group. The inclusion criteria were registered nurses who were currently working in a hospital, informed about the survey, and participated voluntarily. We excluded refresher nurses, nurses from external hospitals who were there to receive standardized training, and nursing student interns.

### Data quality control

To ensure the integrity of the data we collected we placed requirements on the hospital questionnaire distributors before administering the survey. For example, we instructed them that the survey respondents should come from a variety of departments. Additionally, the questionnaire link was only forwarded to their healthcare providers working groups to ensure that the participants were indeed healthcare workers, and we also used the survey question of “job position” to check that a research subject was a direct healthcare provider. Furthermore, we used concise, easy-to-understand phrasing marked some keywords in red so that the topics were more readable. In order to reduce missing data the questionnaires could be submitted only when filled out completely. Finally, to avoid duplicate questionnaires, we limited the number of surveys per IP address to one.

### Questionnaire

A self-designed questionnaire based on relevant policies, guidelines for HIV diagnosis and treatment in China in 2018, and WHO guidelines for HIV post-exposure prophylaxis was used as the primary survey instrument in this study [17, 26]. Experts in this field were also consulted in this regard. A pre-survey questionnaire was formed after revision and improvement. Two hundred questionnaires were then distributed for pre-survey analysis. After collecting and organizing the data, the final version of questionnaire was created according to the feedback from the pre-survey. The questionnaire content validity index (CVI) was 0.87.

This study focuses on three parts: participants’ personal characteristics, the risk assessment of occupational exposure, and administrative policies regarding occupational protection. Personal characteristics included professional title, sex, age, work experience (years), department, and province. For occupational exposure risk assessment, participants were asked whether they wore gloves when attending to patients where there was a risk of blood and body fluids, and whether occupational exposure had been experienced. They were also asked about the average hours worked per day. Participants who had experienced occupational exposures were also asked about the causes and the body parts exposed to blood and body fluids. Questions about administrative policies regarding occupational protection included what PPE was provided, what safety-engineered injection devices were available, and whether nurses received training on occupational exposure. In the Supplement material, questionnaires are provided.

### Data analysis

The data we collected through the online survey platform was exported to Microsoft Excel, checked by two researchers, and then analyzed using IBM SPSS Statistics 23. Descriptive statistics were used to describe the demographic characteristics of the respondents as well as the characteristics of occupational exposure. All variables were categorical variables, so there was no continuous variables. Categorical variables were expressed as frequencies and percentages. Pearson chi-square tests and binary logistic regression were used to examine differences between groups (0 = unexposed vs. 1 = exposed), and we also calculated the *P*-values for each statistical test, as well as the odds ratio (OR) and 95% confidence interval(95%CI). The binary logistic regression analysis examined the associations between various factors and whether occupational exposure occurred. We took *P*-values < 0.05 (for two-tailed tests) to mean that any nonzero differences between groups were statistically significant.

### Ethical approval and consent to participate

Ethical approval to conduct this study was obtained from the Ethics Committee of Xiangya Nursing School, Central South University (E2020128). When study participants clicked on the link to the questionnaire, they were first presented with an informed consent page. They were informed that no harm would come to them as a result of participating in the study and that all responses would be kept confidential. They were entitled to refuse to participate in and were also afforded the opportunity to terminate the questionnaire at any time. All questionnaires were completed anonymously and voluntarily.

## Results

### Population characteristics

A total of 20,791 nurses, comprising 46.0% from the eastern region of China, 30.3% from the western region, 18.3% from the central region, and 5.4% from the northeastern region, participated in this study, as shown in Table 1. Of these, 20,253 were females (97.4%), and 538 were males (2.6%). The majority of nurses were from tertiary hospitals (78.4%), had worked for 10 years or less (62.0%), and had professional title of “primary” (67.8%).

**Table 1:** Demographic characteristics of the respondents (*n* = 20,791)

Characteristics	Participants (n)	Percent (%)	Characteristics	Participants (n)	Percent (%)
**Professional title**			**Work department**		
Primary	14,087	67.8	Internal medicine	9347	45.0
Intermediate	5669	27.3	Surgery	3563	17.1
Associate senior or Senior	1035	5.0	Obstetrics and gynecology	1351	6.5
**Gender**			Pediatrics	1215	5.8
Male	538	2.6	Operating room	754	3.6
Female	20,253	97.4	Emergency	1144	5.5
**Age**			ICU	1268	6.1
18–25	3673	17.7	Nursing	205	1.0
26–30	6769	32.6	Other (paramedical units, blood collection room, injection room, blood dialysis room, etc.)	1944	9.4
31–40	7311	35.2	**Region of hospital**		
41–50	2497	12.0	Eastern	9554	46.0
51–60	541	2.6	Central	3798	18.3
**Work experience (years)**			Western	6309	30.3
≤ 5	6248	30.1	Northeastern	1130	5.4
5–10	6634	31.9	**Hospital level**		
10–20	4942	23.8	Tertiary	16,307	78.4
> 20	2967	14.3	Secondary	4484	21.6

### Epidemiological characteristics of occupational exposure

Over half of the nurses (52.1%; 10,837/20,791) in tertiary/secondary hospitals in China had ever experienced occupational exposure to blood or body fluids, but 34.6% (3747/10,837) did not ever report their exposures to a supervisor/official. The major causes of underreporting were that source patient failed to test positive for infectious pathogens such as HBV, HCV, and HIV (43.6%), that the reporting process was too burdensome (24.6%), and indifferent attitudes towards being infected (16.9%). The characteristics of occupational exposure are displayed in Table 2. Hands (73.9%) were the most common body part that was exposed, and the route of exposure was most frequently percutaneous exposure (66.5%), followed by mucous-membrane exposure (32.2%).

**Table 2:** Characteristics of occupational exposure (*n* = 10,837)

Characteristics	Participants (n)	Percent (%)
**What were the body parts exposed to blood and body fluids in your occupational exposure (multiple-choice question)?**		
Hand	10,599	73.9 (10,599/14348)
Foot	535	3.7 (535/14348)
Eye	2115	14.7 (2115/14348)
Forearm	991	6.9 (991/14348)
Others	108	0.8 (108/14348)
**What were the circumstances of your occupational exposure (multiple-choice question)?**		
Recapping needle	4708	14.6 (4708/32226)
Disposing of discarded sharps	6507	20.2 (6507/32226)
Withdrawing needles	6220	19.3 (6220/32226)
Suturing	1546	4.8 (1546/32226)
Delivering sharps	2037	6.3 (2037/32226)
Being accidentally injured by others	2835	8.8 (2835/32226)
Spattering of blood and secretions	5237	16.3 (5237/32226)
Being scratched by patients	2846	8.8 (2846/32226)
Other	290	0.9 (290/32226)
**What were the exposure routes of your occupational exposure (multiple-choice question)?**		
Percutaneous exposure	10,402	66.5 (10,402/15633)
Mucous-membrane exposure	5031	32.2 (5031/15633)
Other	200	1.3 (200/15633)
**What were the reasons for the occupational exposure (multiple-choice question)?**		
Absent-mindedness during operating	4702	16.6 (4702/28376)
No safety injection tool available	2554	9.0 (2554/28376)
Not using safety injection tool	1200	4.2 (1200/28376)
Chaotic operating circumstances or insufficient light	3380	11.9 (3380/28376)
Improper disposal of sharp objects	6819	24.0 (6819/28376)
Noncompliance with standard practices	3696	13.0 (3696/28376)
Manipulating a needle in an agitated patient	5516	19.4 (5516/28376)
Other	509	1.8 (509/28376)
**Have you ever reported any occupational exposure?**		
Yes	7090	65.4 (7090/10837)
No	3747	34.6 (3747/10837)
**What were the reasons for underreporting (multiple-choice question)?**^**a**^		
Didn’t know the reporting process	266	4.7 (266/5699)
Burdensome reporting process	1404	24.6 (1404/5699)
Source patient failed to test positive for infectious pathogens	2483	43.6 (2483/5699)
Indifferent attitude towards being infected	962	16.9 (962/5699)
Afraid of being criticized	281	4.9 (281/5699)
Fear of discrimination	73	1.3 (73/5699)
Other	230	4.0 (230/5699)
**What amount of stress did you experience after your occupational exposure?**		
Low	315	2.9 (315/10837)
General	2401	22.2 (2401/10837)
High	8121	74.9 (8121/10837)
**If applicable, what were the reasons for your high level of stress after exposure (multiple-choice question)?**^**b**^		
Worry about being infected	8087	49.3 (8087/16395)
Worry about side effects of prophylactic medication	3611	22.0 (3611/16395)
Fear of leadership criticism	1790	10.9 (1790/16395)
Fear of exposure becoming known to family members	2808	17.1 (2808/16395)
Other	99	0.6 (99/16395)

For multiple-choice questions, Percentage = number of people choosing this option / total number of choices

^a^ means *n* = 3747. ^b^ means *n* = 8121

In the exposure group, which had 10,837 members, the most occupational exposures occurred during the disposal of discarded sharps (20.2%), followed by the withdrawal of needles (19.3%), the spattering of blood and secretions (16.3%), and the recapping of needles (14.6%). The most common causes of exposure were improper disposal of sharps (24.0%), manipulating needles in agitated patients (19.4%), absent-mindedness during procedures (16.6%), noncompliance with standard practices (13.0%), and chaotic operating circumstances or insufficient light (11.9%). In addition, 74.9% of nurses said that they were under high stress after their exposure. The most commonly reported reason for this high level of stress was fear of being infected (49.3%), followed by fear of side effects of prophylactic medication (22.0%) and fear of family members knowing about the exposure (17.1%).

### Factors related to occupational exposure to blood and body fluids among nurses

Our univariate analysis using Pearson chi-square tests showed that professional title (χ^2^ = 358.795, *P* < 0.001), gender (χ^2^ = 5.751, *P* = 0.016), age (χ^2^ = 423.150, *P* < 0.001), work experience (χ^2^ = 430.787, *P* < 0.001), work department (χ^2^ = 114.907, *P* < 0.001), region of hospital (χ^2^ = 79.897, *P* < 0.001), hospital level (χ^2^ = 6.108, *P* = 0.013), PPE (χ^2^ = 457.852, *P* < 0.001), safety-engineered injection devices (χ^2^ = 343.524, *P* < 0.001), occupational safety protection training (χ^2^ = 300.860, *P* < 0.001), work hours per day (χ^2^ = 115.050, *P* < 0.001), wearing gloves when making contact with blood or body fluids (χ^2^ = 594.730, *P* < 0.001), following standard prevention procedures (χ^2^ = 423.564, *P* < 0.001), self-evaluation of the risk level of occupational exposure (χ^2^ = 307.221, *P* < 0.001), and a culture of safety (χ^2^ = 535.896, *P* < 0.001) were all statistically associated with whether occupational exposure occurred, as presented in Table 3.

**Table 3:** Univariate analysis for occupational exposure-related factors

Variables	Occupational Exposure n(%)		*P*	OR (95%CI)
	No	Yes		
**Professional title**				
Primary	7379	6708 (47.6%)	< 0.001	1
Intermediate	2205	3464 (61.1%)		1.728 (1.623, 1.840)
Associate senior or Senior	370	665 (64.3%)		1.977 (1.734, 2.255)
**Gender**				
Male	285	253 (47.0%)	0.016	1
Female	9669	10,584 (52.3%)		1.233 (1.039, 1.464)
**Age**				
18–25	2241	1432 (39.0%)	< 0.001	1
26–30	3374	3395 (50.2%)		1.575 (1.451, 1.709)
31–40	3128	4183 (57.2%)		2.093 (1.930, 2.269)
41–50	973	1524 (61.0%)		2.451 (2.209, 2.720)
51–60	238	303 (56.0%)		1.992 (1.660, 2.391)
**Work experience (years)**				
≤ 5	3640	2608 (41.7%)	< 0.001	1
5–10	3080	3554 (53.6%)		1.610 (1.502, 1.727)
10–20	2038	2904 (58.8%)		1.989 (1.844, 2.145)
> 20	1196	1771 (59.7%)		2.067 (1.891, 2.259)
**Work department**				
Internal medicine department	4382	4965 (53.1%)	< 0.001	1
Surgery department	1650	1913 (53.7%)		1.023 (0.947, 1.106)
Obstetrics and gynecology department	725	626 (46.3%)		0.762 (0.680, 0.854)
Pediatrics department	603	612 (50.4%)		0.896 (0.795, 1.010)
Operating room	266	488 (64.7%)		1.619 (1.387, 1.890)
Emergency department	546	598 (52.3%)		0.967 (0.855, 1.093)
ICU	707	561 (44.2%)		0.700 (0.622, 0.788)
Nursing department	88	117 (57.1%)		1.173 (0.887, 1.552)
Others (paramedical units, blood collection room, injection room, blood dialysis room and so on)	987	957 (49.2%)		0.856 (0.776, 0.944)
**Region of hospital**				
Eastern	4815	4739(49.6%)	< 0.001	1
Central	1594	2204(58.0%)		1.405(1.302, 1.516)
Western	3030	3279(52.0%)		1.100(1.032, 1.172)
Northeastern	515	615(54.4%)		1.213(1.072, 1.373)
**Hospital level**				
Tertiary	7734	8573(52.6%)	0.013	1
Secondary	2220	2264(50.5%)		0.920(0.861, 0.983)
**Do you follow standard prevention strategies during working?**				
Yes	8675	8525(49.6%)	< 0.001	1
No	540	1506 (73.6%)		2.838 (2.561, 3.145)
No knowledge of what standard prevention practices are	739	806 (52.2%)		1.110 (1.000, 1.232)
**How many hours do you work per day?**				
8 hours	5602	5293 (48.6%)	< 0.001	1
> 8 hours	4352	5544 (56.0%)		1.348 (1.277, 1.424)
**Did you wear gloves when attending to patients that posed a risk for blood and body fluid exposure?**				
Yes	8277	7437 (47.3%)	< 0.001	1
Occasionally	1467	2929 (66.6%)		2.222 (2.072, 2.383)
No	210	471 (69.2%)		2.496 (2.115, 2.946)
**What do you think the risk level of occupational exposure at your workplace is**?				
Low	453	253 (35.8%)	< 0.001	1
General	2614	1961 (42.9%)		1.343 (1.139, 1.584)
High	6887	8623 (55.6%)		2.242 (1.916, 2.623)
**How do you rate the awareness of occupational safety protection of the people you work with?**				
Strong	5311	4111 (43.6%)	< 0.001	1
General	4317	6023 (58.2%)		1.802 (1.704, 1.907)
Not strong enough	326	703 (68.3%)		2.786 (2.428, 3.197)
**How many kinds of PPE does your hospital provide to healthcare providers?**				
9–10	2237	1642 (42.3%)	< 0.001	1
7–8	2527	2288 (47.5%)		1.234 (1.133, 1.343)
5–6	2441	2674 (52.3%)		1.492 (1.372, 1.623)
3–4	1730	2239 (56.4%)		1.763 (1.612, 1.928)
1–2	1019	1994 (66.2%)		2.666 (2.415, 2.943)
**How many kinds of safety-engineered injection devices are available to nurses in your hospital?**				
5–6	3015	2461 (44.9%)	< 0.001	1
3–4	3349	3114 (48.2%)		1.139 (1.060, 1.224)
1–2	3590	5262 (59.4%)		1.796 (1.678, 1.922)
**What is the interval between training sessions related to occupational safety protection in your hospital?**				
1 month	2562	1925 (42.9%)	< 0.001	1
3 months	2226	2140 (49.0%)		1.279 (1.177, 1.391)
6 months	1410	1628 (53.6%)		1.537 (1.401, 1.686)
1 year	1533	1899 (55.3%)		1.649 (1.507, 1.803)
> 1 year, none, or no idea	2223	3245 (59.3%)		1.943 (1.793, 2.105)

In addition, we used binary logistic regression analysis to examine the associations between several risk factors and whether occupational exposure occurred. These results are presented in Table 4. The risk of occupational exposure for operating room nurses (OR = 1.642, 95% CI = 1.395, 1.932) is 1.642 times greater than internal medicine nurses, and the risk of occupational exposures in the central (OR = 1.311, 95% CI = 1.208, 1.422) region of China is higher than in the eastern region. Furthermore, nurses who work over 8 hours per day (OR = 1.199, 95% CI = 1.130, 1.272) have 1.199 times the risk of occupational exposure compared to those who do not.

**Table 4:** Multivariate analysis of occupational exposure-related factors

Variables	B	OR	OR (95%CI)	*P*
**Work experience (years)**				
≤ 5	Ref	1		
5–10	0.435	1.546	(1.436, 1.664)	< 0.001
10–20	0.686	1.986	(1.831, 2.154)	< 0.001
> 20	0.852	2.344	(2.124, 2.588)	< 0.001
**Work department**				
Internal medicine	Ref	1		
Surgery	0.013	1.014	(0.934, 1.100)	0.748
Obstetrics and gynecology	−0.193	0.825	(0.731, 0.931)	0.002
Pediatrics	−0.037	0.964	(0.849, 1.095)	0.572
Operating room	0.496	1.642	(1.395, 1.932)	< 0.001
Emergency	−0.059	0.943	(0.828, 1.074)	0.374
ICU	−0.311	0.733	(0.647, 0.830)	< 0.001
Nursing	0.420	1.522	(1.122, 2.066)	0.007
Other (paramedical units, blood collection room, injection room, blood dialysis room, etc.)	− 0.090	0.914	(0.823, 1.016)	0.095
**Region of hospital**				
Eastern	Ref	1		
Central	0.271	1.311	(1.208, 1.422)	< 0.001
Western	0.022	1.022	(0.954, 1.096)	0.531
Northeastern	0.030	1.031	(0.904, 1.176)	0.651
**Hospital level**				
Tertiary	Ref	1		
Secondary	−0.073	0.930	(0.865, 1.000)	0.049
**Do you follow standard prevention strategies during working?**				
Yes	Ref	1		
No	0.736	2.088	(1.872, 2.330)	< 0.001
No knowledge of what standard prevention practices are	−0.077	0.926	(0.828, 1.036)	0.180
**How many hours do you work per day?**				
8 hours	Ref	1		
> 8 hours	0.181	1.199	(1.130, 1.272)	< 0.001
**What do you think the risk level of occupational exposure at your workplace is?**				
Low	Ref	1		
General	0.371	1.449	(1.211, 1.735)	< 0.001
High	0.846	2.331	(1.958, 2.775)	< 0.001
**How about the awareness of occupational safety and protection of people around you?**				
Strong	Ref	1		
General	0.391	1.478	(1.390, 1.571)	< 0.001
Not strong enough	0.630	1.877	(1.622, 2.173)	< 0.001
**How many kinds of PPE does your hospital provide to healthcare providers?**				
9–10	Ref	1		
7–8	0.053	1.054	(0.961, 1.157)	0.266
5–6	0.234	1.264	(1.151, 1.387)	< 0.001
3–4	0.346	1.413	(1.278, 1.562)	< 0.001
1–2	0.666	1.947	(1.740, 2.178)	< 0.001
**How many kinds of safety-engineered injection devices are available to nurses in your hospital?**				
5–6	Ref	1		
3–4	−0.032	0.968	(0.894, 1.049)	0.429
1–2	0.243	1.275	(1.179, 1.379)	< 0.001
**What is the interval between training sessions related to occupational safety protection in your hospital?**				
1 month	Ref	1		
3 months	0.095	1.100	(1.007, 1.201)	0.035
6 months	0.188	1.207	(1.094, 1.332)	< 0.001
1 year	0.155	1.168	(1.061, 1.286)	0.002
> 1 year, none, or no idea	0.300	1.350	(1.236, 1.474)	< 0.001

The occupational exposure risk from providing 1–2 types of PPE is 1.947 times that of providing 9–10 types of PPE. Likewise, the occupational exposure risk of providing 1–2 types of safety-engineered injection devices is 1.275 times of that of providing 5–6 types. The types of hospital-provided PPE and safety-engineered injection devices are shown in Table 5. Additionally, nurses with occupational safety protection training frequencies of less than 1 time per year have 1.350 times the risk of occupational exposure of those who received training at least once per month. Nurses who do not follow standard prevention practices have 2.088 times the risk of occupational exposure of those who do.

**Table 5:** Types of hospital-provided PPE and safety-engineered injection devices

Questions	Answers	Participants (n)	Percent (%)
**What PPE does your hospital provide to healthcare providers?**			
Disposable surgical mask	No	2057	9.9
	Yes	18,734	90.1
N95 protective mask	No	9856	47.4
	Yes	10,935	52.6
Protective screen mask	No	13,493	64.9
	Yes	7298	35.1
Disposable gloves	No	1677	8.1
	Yes	19,114	91.9
Needle-stick-resistant surgical gloves	No	13,229	63.6
	Yes	7562	36.4
Protective glass	No	7051	33.9
	Yes	13,740	66.1
Isolation gowns	No	5083	24.4
	Yes	15,708	75.6
Disposable gowns	No	8230	39.6
	Yes	12,561	60.4
Shoe covers	No	8140	39.2
	Yes	12,651	60.8
Other	No	20,605	99.1
	Yes	186	0.9
**What safety-engineered injection devices are available to nurses in your hospital?**			
Safety indwelling needle	No	2234	10.7
	Yes	18,557	89.3
Safety blood collection needle	No	7351	35.4
	Yes	13,440	64.6
Safety arterial blood collection needle	No	10,229	49.2
	Yes	10,562	50.8
Safety syringe	No	11,562	55.6
	Yes	9229	44.4
Needle-less infusion connectors	No	10,357	49.8
	Yes	10,434	50.2
Other	No	20,100	96.7
	Yes	691	3.3

## Discussion

Occupational exposure to blood and body fluids in Chinese registered nurses is still common, and the rate of exposure under-reporting is high. Work experience, work department, region of hospital, hospital level, working hours per day, following standard prevention practices, perceived level of risk of occupational exposure in the workplace, awareness of occupational safety and the protection of the people around you, number of types of PPE provided by the hospital, number of types of safety-engineered injection devices provided by the hospital, and frequency of training related to occupational safety can all impact the occurrence of nurses’ occupational exposure to blood and body fluids.

There is a high prevalence of occupational exposure to blood and body fluids among nurses in China, and this result is consistent with previous studies [27, 28]. The most common route of exposure is percutaneous (needlestick, sharp injuries, broken skin, etc.), accounting for about 2/3 of all incidents, followed by mucous-membrane exposure, which accounts for about 1/3. Globally, there is a high incidence of percutaneous injury among healthcare providers who directly care for patients that increases their risk of infection from bloodborne viruses such as HBV, HCV, and HIV [29]. In this study we found that disposing of discarded sharps and withdrawing needles were the most common circumstances associated with occupational exposure, and the most common cause of occupational exposure was disposing of sharp objects without following the correct procedures, which is also similar to previous findings [4, 30]. However, most current training courses for nursing technical procedures do not emphasize sharp injury prevention enough [31]. This suggests that such training content should be added to future textbooks and training courses.

In addition, this study showed that the more comprehensive the provision of safety-engineered sharps, the lower the occurrence of occupational exposure, which agrees with the results of a multicenter study in Japan that showed that the application of safety-engineered syringes significantly reduced the incidence of needlestick injuries [32]. Moreover, a meta-analysis also demonstrated that sharp injury prevention syringes lowered the incidence of needlestick injuries [33]. As previously mentioned, in 2015, the WHO appealed for worldwide use of safety-engineered syringes by 2020 [19]. However, safety blood collection needles, safety arterial blood collection needles, safety syringes, and needleless infusion connectors remain scarce, especially safety syringes. Less than 1/2 of the nurses in this study had received these. Compared to secondary hospitals, the tertiary hospitals in our study provided more comprehensive safety injection tools. Despite this, only about half of the nurses in the entire study had access to safety arterial blood collection needles. Hence, promoting the use of safety-engineered sharps in hospitals should help to reduce the occurrence of exposure to blood and body fluids.

Our results also show that receiving adequate education and training on occupational exposure and standard prevention practices is beneficial for reducing the risk of exposure. Over 1/10 of the occupational exposures in this study occurred due to noncompliance with standard practices, and about 1/4 of the nurses do not wear or only occasionally wear gloves during encounters with high-risk patients. Thus, nursing administrators in China may want to strengthen education and training on occupational exposure and standard practices and increase the frequency of training sessions.

One previous study in the Netherlands showed that there were some safety engineered devices related to needle stick injuries, such as nadroparin calcium needles and infusion needles [34]. The top two causes of safety engineered devices related to needlestick injuries were needles being unsafely disposed and problems with safety engineered devices [34]. Furthermore, a meta-analysis showed that safeguarded intravenous cannulas reduced the incidence of needlestick injuries but at the cost of increased incidence of blood exposure [35]. These results may be attributed to the fact that these new devices are more difficult for healthcare providers to use [35]. Therefore, in addition to the knowledge of standard prevention practices, the skills of how to operate new safety-engineered syringes are quite important and should be trained regularly.

Additionally, absent-mindedness during procedures is a common cause of occupational exposure. Nursing is a high-intensity profession that requires nurses to focus intently, sometimes for long periods of time. Long working hours can lead to a loss of concentration that can increase the risk of accidents including occupational exposure [36–38]. A study in Taiwan showed that nurses who work 41–50 hours per week and > 50 hours per week had 1.17 times and 1.51 times the risk of needlestick injuries, respectively, compared to those who worked no more than 40 hours per week [36]. This implies that the problem of how to ensure nurses on duty concentrate on the task at hand needs to be addressed directly.

Apart from that, as with previous studies [10, 28], the present study also showed that the self-reporting rate of occupational exposure among nurses is low, with a rate reporting of less than 2/3. The major reason given for this was that bloodborne pathogens had failed to be detected in the source patients. Additionally, the reporting procedure was often described as burdensome, which was the second most common reason given for not reporting. This suggests that a simpler reporting procedure should be applied. Some participants in this study were even unaware of the reporting process entirely. Similarly to a previous study, some nurses expressed that they did not know where to report such incidents [39]. In addition, some participants thought they would not be infected even after the exposure, and some expressed fear of being criticized and facing discrimination. Reporting the occupational exposure is of great importance for both PEP and for the diagnosis of potential infections as early as possible [40, 41]. With earlier and more reporting, psychological stress of the exposed nurses could potentially be reduced. This study showed that over 70% of exposed nurses had high stress after exposure, especially female nurses who had worked for less than 10 years. Thus, it makes sense for hospitals to pay proper attention to exposure incidents, to encourage staff to report every one of them, and to provide prompt counseling to the exposed.

### Limitations

Despite it merits, this study still has several limitations. First, this study was a recall survey of whether nurses had experienced occupational exposure, and recall bias may not have been entirely excluded. But the frequency of occupational exposure was not investigated in this study, and recall bias was small. Finally, primary hospitals were not analyzed due to the small proportion of nurses (about 1.1%) from primary hospitals.

### Recommendations for future research

The impact of the use of safety engineered devices on occupational exposure should be further explored in future work, along with the development of devices that are more conducive to both nurse and patient safety.

### Clinical implications for nursing managers and policymakers

Based on the results of this study, we recommended strengthening precautionary measures aimed at preventing occupational exposure to blood and body fluids, including occupational safety training, training on the use of new safety engineered devices, provision of adequate PPE, and creating a culture of safety in the workplace. We also recommend conducting clinical trials and recording factors that may cause occupational exposure during use before formally introducing safety engineered devices, and implementing policies that schedule shifts in a way that ensures adequate rest for nurses in order to reduce occupational exposure due to absent-mindedness. Finally, we recommend simplifying the process of reporting occupational exposure, and strengthening post-exposure support resources.

## Conclusions

Occupational exposure to blood and body fluids in Chinese registered nurses remains common, and the rate of exposure underreporting is also quite high. Hours worked per day, not following standard prevention practices, awareness of occupational safety, number and types of PPE provided by the hospital, number and types of safety-engineered injection devices provided by the hospital, and frequency of safety training were all found to have an impact on nurses’ occupational exposure. We suggest that implementing engineered sharps injury prevention devices, strictly following exposure prevention practices, giving sufficient education and training to healthcare personnel, and developing exposure reporting policies can reduce the rates of both the occurrence of exposure and of not reporting exposure when it happens.

## Supplementary Information

**Additional file 1.**

## Data Availability

The datasets used and/or analysed during the current study available from the corresponding author on reasonable request.

## Abbreviations

HBV: Hepatitis B Virus
HCV: Hepatitis C Virus
HIV: Human Immunodeficiency Virus
WHO: World Health Organization
PEP: Post-Exposure Prophylaxis
ILO: International Labor Organization
PPE: Personal Protective Equipment
CVI: content validity index
OR: Odds Ratio
CI: Confidence Interval

## References

[1] Yasin J, Fisseha R, Mekonnen F, Yirdaw K (2019). Occupational exposure to blood and body fluids and associated factors among health care workers at the University of Gondar Hospital, Northwest Ethiopia. Environ Health Prev Med.

[2] Sahiledengle B, Tekalegn Y, Woldeyohannes D, Quisido BJE (2020). Occupational exposures to blood and body fluids among healthcare workers in Ethiopia: a systematic review and meta-analysis. Environ Health Prev Med.

[3] Jahic R, Piljic D, Porobic-Jahic H, Custović A, Petrovic J, Piljic D (2018). Epidemiological characteristics of the accidental exposures to blood-borne pathogens among Workers in the Hospital. Med Arch.

[4] Song XZ, Fang X, Ding J, Jin L, You J (2020). Investigation of 603 medical staff occupational exposure with blood-borne pathogens. Chin J Industrial Hygiene Occupational Dis.

[5] Tavoschi L, Mason L, Petriti U, Bunge E, Veldhuijzen I, Duffell E (2019). Hepatitis B and C among healthcare workers and patient groups at increased risk of iatrogenic transmission in the European Union/European economic area: a systematic review. J Hosp Infect..

[6] World Health Organization. The world health report 2002. https://www.who.int/whr/2002/en/. Accessed 25 Feb 2021.

[7] Maida CM, Aprea L, Calamusa G, Campisi F, Favaro D, Russo Fiorino G, et al. Blood and body fluids exposure of healthcare workers in a university hospital of Palermo, Italy: a fourteen years long surveillance. Ann Ig. 2020;32(6). 10.7416/ai.2020.2380. 10.7416/ai.2020.2380 33029611

[8] Lin J, Gao X, Cui Y, Sun W, Shen Y, Shi Q, Chen X, Hu B. A survey of sharps injuries and occupational infections among healthcare workers in Shanghai. Ann Transl Med 2019;7(22):678. 10.21037/atm.2019.10.42. 10.21037/atm.2019.10.42 PMC6944561 31930079

[9] Garus-Pakowska A, Górajski M (2019). Behaviors and attitudes of polish health care workers with respect to the hazards from blood-borne pathogens: a questionnaire-based study. Int J Environ Res Public Health.

[10] Kasatpibal N, Whitney JD, Katechanok S, Ngamsakulrat S, Malairungsakul B, Sirikulsathean P, Nuntawinit C, Muangnart T (2016). Practices and impacts post-exposure to blood and body fluid in operating room nurses: a cross-sectional study. Int J Nurs Stud.

[11] Jeong JS, Son HM, Jeong IS, Son JS, Shin KS, Yoonchang SW, Jin HY, Han SH, Han SH (2016). Qualitative content analysis of psychologic discomfort and coping process after needlestick injuries among health care workers. Am J Infect Control.

[12] Shi Y, Xue H, Ma Y, Wang L, Gao T, Shi L, Wang Y, Cui M, Wang C, Yang X, Liu M, Fan L, Yan G (2020). Prevalence of occupational exposure and its influence on job satisfaction among Chinese healthcare workers: a large-sample, cross-sectional study. BMJ Open.

[13] World Health Organization. World Patient Safety Day 2020. https://www.who.int/campaigns/world-patient-safety-day/2020. Accessed 19 Apr 2021.

[14] Wang D, Ye Y, Zheng Q (2020). Cost of blood and body fluid occupational exposure Management in Beijing, China. Int J Environ Res Public Health.

[15] Zhao F, Zhang M, Xuan J, Mo Y, Huang J, Liu Z, Zhou L, Xu Y, Guo X (2019). Burden of insulin injection-related needlestick injuries in mainland China-prevalence, incidence, and healthcare costs. Int J Nurs Stud.

[16] Kuhar DT, Henderson DK, Struble KA, et al. Updated U.S. Public Health Service guidelines for the management of occupational exposures to HIV and recommendations for postexposure prophylaxis. https://stacks.cdc.gov/view/cdc/20711. Accessed 19 Apr 2021.

[17] AIDS and Hepatitis C Professional Group, Society of Infectious Diseases, Chinese Medical Association; Chinese Center for Disease Control and Prevention. Chinese guidelines for diagnosis and treatment of HIV/AIDS (2018). Chin J Intern Med. 2018;57(12):867–884.

[18] Moorman AC, de Perio MA, Goldschmidt R, Chu C, Kuhar D, Henderson DK, Naggie S, Kamili S, Spradling PR, Gordon SC, Russi MB, Teshale EH (2020). Testing and clinical Management of Health Care Personnel Potentially Exposed to hepatitis C virus - CDC guidance, United States, 2020. MMWR Recomm Rep.

[19] World Health Organization. WHO calls for worldwide use of "smart" syringes. https://www.who.int/news/item/23-02-2015-who-calls-for-worldwide-use-of-smart-syringes. Accessed 23 Feb 2021.

[20] International labour Organization. Nurse, occupational health: International Hazard Datasheets on Occupation. https://www.ilo.org/safework/cis/WCMS_192435/lang%2D%2Den/index.htm. Accessed 19 Feb 2021.

[21] King KC, Strony R. Needlestick. https://www.ncbi.nlm.nih.gov/books/NBK493147/. Accessed 19 Feb 2021.

[22] Zhu B, Fan H, Xie B, Su R, Zhou C, He J (2020). Mapping the scientific research on healthcare Workers' occupational health: a bibliometric and social network analysis. Int J Environ Res Public Health.

[23] Zhang Yan-hua, Bai Jia-wei, Zhou Ying-shun. Characteristics of occupational exposure to blood-borne pathogens in a hospital in Southwest China from 2015 to 2019. Chin J Infect Control. 2020;19(12):1054–1058. 10.12138/j.issn.1671-9638.20206169.

[24] Gao X, Hu B, Suo Y, Lu Q, Chen B, Hou T, Qin J, Huang W, Zong Z. A large-scale survey on sharp injuries among hospital-based healthcare workers in China. Sci Rep 2017;7:42620. 10.1038/srep42620. 10.1038/srep42620 PMC5311998 28205607

[25] Cui Z, Zhu J, Zhang X, Wang B, Li X (2018). Sharp injuries: a cross-sectional study among health care workers in a provincial teaching hospital in China. Environ Health Prev Med.

[26] Ford N, Mayer K. World Health Organization guidelines on Postexposure prophylaxis for HIV: recommendations for a public health approach. Clin Infect Dis. 2015:S161–4. 10.1093/cid/civ068. 10.1093/cid/civ068 25972497

[27] Belachew YB, Lema TB, Germossa GN, Adinew YM (2017). Blood/body fluid exposure and needle stick/sharp injury among nurses working in public hospitals; Southwest Ethiopia. Front Public Health.

[28] Yi Y, Yuan S, Li Y, Mo D, Zeng L (2018). Assessment of adherence behaviors for the self-reporting of occupational exposure to blood and body fluids among registered nurses: a cross-sectional study. PLoS One.

[29] Auta A, Adewuyi EO, Tor-Anyiin A, Edor JP, Kureh GT, Khanal V, Oga E, Adeloye D (2018). Global prevalence of percutaneous injuries among healthcare workers: a systematic review and meta-analysis. Int J Epidemiol.

[30] Hui LI, Xiu-wen CHEN, Cao PENG, Yue-jiao WANG, Yun-xia LI, Li ZENG, Ming YANG, Su-e YUAN (2017). Status of needlestick injuries among nurses in China during venous blood sampling. Chin J Infect Control.

[31] Li Xiaohan, Shang Shaomei. Basic nursing, 6th Ed. Beijing: People's Medical Publishing House; 2017.

[32] Fukuda H, Yamanaka N (2016). Reducing needlestick injuries through safety-engineered devices: results of a Japanese multi-Centre study. J Hosp Infect..

[33] Harb AC, Tarabay R, Diab B, Ballout RA, Khamassi S, Akl EA (2015). Safety engineered injection devices for intramuscular, subcutaneous and intradermal injections in healthcare delivery settings: a systematic review and meta-analysis. BMC Nurs.

[34] Schuurmans J, Lutgens SP, Groen L, Schneeberger PM (2018). Do safety engineered devices reduce needlestick injuries?. J Hosp Infect.

[35] Ying GU, Yan HU, Feng ZHANG (2015). The protection effect of safeguarded intravenous cannulas on healthcare personnel occupational exposure: a meta-analysis. Chin J Nurs.

[36] Lo WY, Chiou ST, Huang N, Chien LY (2016). Long work hours and chronic insomnia are associated with needlestick and sharps injuries among hospital nurses in Taiwan: a national survey. Int J Nurs Stud.

[37] Ropponen A, Koskinen A, Puttonen S, Härmä M (2019). Exposure to working-hour characteristics and short sickness absence in hospital workers: a case-crossover study using objective data. Int J Nurs Stud.

[38] Auta A, Adewuyi EO, Tor-Anyiin A, Aziz D, Ogbole E, Ogbonna BO, Adeloye D (2017). Health-care workers' occupational exposures to body fluids in 21 countries in Africa: systematic review and meta-analysis. Bull World Health Organ.

[39] Rasweswe MM, Peu MD (2020). Occupational exposure to blood and body fluids and use of human immunodeficiency virus post-exposure prophylaxis amongst nurses in a Gauteng province hospital. Health SA.

[40] Dong Y, Li F, Li J, Li R, Wang Q (2020). Multicenter cross-sectional study on the reporting status and influencing factors of needlestick injuries caused by insulin injection devices among nurses in Peking, China. Am J Infect Control.

[41] Riddell A, Kennedy I, Tong C (2015). Management of sharps injuries in the healthcare setting. BMJ..

