# Supplementary material for: Prevalence and influence factors of occupational exposure to blood and body fluids in registered Chinese nurses: a national cross-sectional study
Source: BMC Nurs. 2022 Nov 4;21:298. doi: 10.1186/s12912-022-01090-y (PMC9636689; doi:10.1186/s12912-022-01090-y)
Supplement: Supplementary file 1 — Additional file 1. [file 12912_2022_1090_MOESM1_ESM.docx]

**Status Survey on Occupational Exposure in Nurses**

**PART ONE Demographic characteristics**

1. What is your professional title

1. Primary
2. Intermediate
3. Associate senior or Senior

2. What gender are you

1. Male
2. Female

3. How old are you

1. 18-25
2. 26-30
3. 31-40
4. 41-50
5. 51-60

4. How long have you been working in the clinic

1. ≤5
2. 5-10
3. 10-20
4. >20

5. What is your professional cadre?

1. Registered nurses
2. Doctors
3. Medical support personnel
4. Nursing managers
5. Other

6. What is your work department

1. Internal medicine department
2. Surgery department
3. Obstetrics and gynecology department
4. Pediatrics department
5. Infection department
6. Operating room
7. Emergency department
8. ICU
9. Auxiliary department
10. Nursing department
11. Other

7. Where is your hospital located [Fill in the blank]

8. What is your hospital level

1. Tertiary
2. Secondary
3. Primary

9. What is the type of your hospital

1. General hospitals
2. Specialized hospitals for infectious diseases
3. Other

10. What is the nature of your hospital

1. Public hospitals
2. Private hospitals
3. Other

**PART TWO Knowledge about occupational exposure**

1. Do you know what standard prevention is

1. No (Jump to question 3)
2. Yes

2. Can you practice following standard prevention strategies during working?

1. No
2. Yes

3. Which processes for handling blood-borne occupational exposure do you know [multiple-choice question]

1. Process for handling occupational exposure to HIV
2. Process for handling occupational exposure to HBV
3. Process for handling occupational exposure to HCV
4. Process for handling occupational exposure to syphilis

4. What is the preferable time to start Post Occupational Exposure Prophylaxis against HIV

1. Within 2 hours
2. Within 4 hours
3. Within 24 hours
4. Within 72 hours
5. No idea

5. The maximum delay to take Post Occupational Exposure Prophylaxis against HIV

1. Within 2 hours
2. Within 4 hours
3. Within 24 hours
4. Within 72 hours
5. No idea

6. Which is the correct way to treat a wound after needle stick exposure

1. Rinse with running water, push the wound from the proximal end to the distal end, and then disinfect it.
2. Push the wound from the distal end to the proximal end, rinse with running water, then disinfect it.
3. Disinfect the wound,rinse with running water, and then push it from the distal end to the proximal end.
4. No treatment, as long as it does not hurt
5. Others

7. Which is the correct way to treat a wound after mucous-membrane exposure

1. No need to handle it
2. Rinse thoroughly with normal saline or distilled water
3. Rinse thoroughly with normal saline or distilled water,then apply antibiotic eye drops for prevention
4. Other

**PART THREE Occupational exposure risk assessment**

1. How many hours do you work per day?

1. 8 hours
2. 8-10 hours
3. 10-12 hours
4. over 12 hours

2. What do you think the risk level of occupational exposure at your workplace is

1. Low
2. General
3. High

3. Did you wear gloves when attending to patients that posed a risk for blood and body fluid exposure?

1. No
2. Occasionally
3. Yes (Jump to question 5)

4. If not, what were the reasons [multiple-choice question]

1. Too busy
2. No awareness of wearing gloves
3. Inconvenient to operate with gloves
4. Reducing departmental costs
5. Other

5. Have you ever experience occupational exposures to blood and body fluids

1. No (Jump to question 6)
2. Yes

5.1 What were the body parts exposed to blood and body fluids in your occupational exposure [multiple-choice question]

1. Hand
2. Foot
3. Eye
4. Forearm
5. Other

5.2 Have you ever reported any occupational exposure

1. Yes (Jump to question 5.4)
2. No

5.3 If not, what were the reasons [multiple-choice question]

1. Don't know the reporting process
2. Burdensome reporting process
3. Source patient failed to detect with infectious pathogens
4. Indifferent attitude towards being infected
5. Afraid of being criticized
6. Fear of discrimination
7. Other

5.4 What were the reasons for occupational exposures [multiple-choice question]

1. Absent-mindedness during operating
2. No safety injection tool available
3. Not using safety injection tool
4. Chaotic operating circumstances or insufficient light
5. Improper disposal of sharp objects
6. Noncompliance with standard practices
7. Manipulating a needle in an agitated patient
8. Other

5.5 What were the circumstances of your occupational exposure [multiple-choice question]

1. Recapping needle
2. Disposing of discarded sharps
3. Withdrawing needles
4. Suturing
5. Delivering sharps
6. Being accidentally injured by others
7. Spattering of blood and secretions
8. Being scratched by patients
9. Other

5.6 What were the exposure routes of your occupational exposure [multiple-choice question]

1. Percutaneous exposure
2. Mucous-membrane exposure
3. Other

6. Were baseline blood testing done on exposed individuals within 24 hours in your hospital

1. No
2. Yes(Jump to question 7)

6.1 If not, what were the reasons [multiple-choice question]

1. Healthcare workers were not aware of the baseline testing
2. Hospitals were not equipped to do baseline testing
3. Higher costs were paid by the exposed individual
4. Other

7. Who paid for the cost of baseline testing

1. The exposed individual
2. The hospital
3. The exposed individual was responsible for the rest except for the portion paid by Medicare
4. The hospital was responsible for the rest except for the portion paid by Medicare
5. Others

8. What amount of stress did you experience after your occupational exposure?

1. Low
2. General
3. High

9. If applicable, what were the reasons for your high level of stress after exposure ? [multiple-choice question]

1. Worried about being infected
2. Worried about side effects of prophylactic medication
3. Fear of leadership criticism
4. Fear of being known by family members
5. Other

**PART FOUR Occupational Exposure Support and Assurance System**

1. What Personal Protective Equipment did your hospital provide for healthcare providers [multiple-choice question]

1. Disposable surgical mask
2. N95 protective mask
3. Protective screen mask
4. Disposable gloves
5. Needle-stick resistant surgical gloves
6. Protective glass
7. Isolation gowns
8. Disposable gowns
9. Shoe covers
10. Other

2. What safety-engineered injection device were available to nurses in your hospital [multiple-choice question]

1. Safety indwelling needle
2. Safety blood collection needle
3. Safety arterial blood collection needle
4. Safety syringe
5. Needle-less infusion connectors
6. Other

3. Was there a comprehensive occupational exposure management process in your hospital

1. No
2. Yes

4. Where did the exposed individuals receive the post-occupational exposure assessments and prophylactic medication in your hospital

1. Own hospital
2. Other tertiary general hospitals
3. Specialized hospitals for infectious diseases
4. Other

5. Did your hospital conduct annual training for all staff on occupational exposure?

1. No (Jump to question 6)
2. Yes

5.1 How often did you receive a training related to occupational safety protection in your hospital?

1. No idea
2. Once a month
3. Once every 3 months
4. Once every 6 months
5. Once a year
6. Less than once a year

5.2 What were the ways in which occupational exposure trainings were carried out in your hospital [multiple-choice question]

1. Self-learning relevant norms
2. Organize study by department,such as reading the norms
3. Personnel from the hospital infection control center went to the department to gave lectures
4. Lectures are held face-to-face throughout the hospital
5. External professional lectures
6. Other

6. Who do you think need to be strengthened training to reduce the occurrence of occupational exposures in hospitals

1. New Staff
2. Staff with 1-5 years of work experience
3. Staff with over 5 years of work experience
4. Staff who undertake teaching
5. Nursing managers

7. Which department do you think is the most appropriate to undertake training on occupational exposure

1. Nursing department
2. Hospital infection control center
3. Medical department
4. Other

8. Do you think it is necessary to have a unified approach to occupational exposure management and to set relevant norms and standards applicable to all hospitals in the country?

1. No
2. Yes

9. How about the awareness of occupational safety protection of people around you

1. Not strong enough
2. General
3. Strong

10. Who did pay for the cost of post occupational exposure prophylaxis

1. The exposed individual
2. The hospital
3. The exposed individual was responsible for the rest except for the portion paid by Medicare.
4. The hospital was responsible for the rest except for the portion paid by Medicare.

11. For occupational exposure, what do you care more about [multiple-choice question]

1. Occupational exposure protection
2. Post-exposure management and follow-up
3. Psychological intervention and support after occupational exposure
4. Compensation and indemnification after occupational exposure
5. Other

**PART FIVE Compensation and Indemnification after Occupational Exposure**

1. Was there a mechanism for compensation and indemnification after occupational exposure in your hospital

1. No
2. Yes
3. No ides

2. What compensation were available to healthcare workers in your hospital after occupational exposure [multiple-choice question]

1. Paid vacation
2. Adjustment work position
3. Free examination, follow-up and medication
4. Financial compensation
5. Visits from hospitals and labor unions
6. Psychological support and intervention
7. Treat as a work-related injury
8. Other

3. Do you think it is necessary/important to have a unified mechanism to compensation management after occupational exposure

1. No
2. Yes
3. No idea

4. Which department do you think should manage the compensation and indemnification mechanism after occupational exposure [multiple-choice question]

1. National Health Commission
2. Regional Health Commission
3. Chinese Nursing Association
4. Regional Nursing Association
5. The hospitals
6. Other

Thanks for your support！
